# Combination of Immunohistochemistry and Ploidy Analysis to Assist Histopathological Diagnosis of Molar Diseases

**DOI:** 10.4137/cpath.s601

**Published:** 2008-03-19

**Authors:** M.C. Osterheld, L. Caron, P. Chaubert, K. Meagher-Villemure

**Affiliations:** Institut Universitaire de Pathologie Rue du Bugnon 25 1011 Lausanne-CHUV

**Keywords:** hydatidiform mole, complete mole, partial mole, hydropic abortion, trophoblastic disease

## Abstract

**Background:**

Differential diagnosis between hydropic abortion, partial mole and complete mole is still a challenge for pathologists but really important for patient management.

**Material and Method:**

In this study, we have evaluated 111 products of conception from the first trimester. Histological analysis was made according to the main diagnostic histopathological features described in the literature and the cases were categorized in hydropic abortus (HA), partial mole (PM) and complete mole (CM). Immunohistochemistry was performed using monoclonal antibody against p57^kip^ protein a putative paternally imprinted inhibitor gene and DNA ploidy was analysed in all cases by image cytometry.

**Results:**

All 23 HAs presented a diploid DNA content and were p57^kip2^ positive. From the 28 CMs, 12 cases (43%) were diploid and 16 cases (57%) were tetraploid but no expression of p57^kip2^ was found with positive internal controls. From the 60 PMs, 58 cases were positive for p57^kip2^ expression and 53 cases (88%) were triploid, 6 cases (10%) tetraploid and 1 case (2%) diploid.

**Conclusion:**

This study on 111 cases of early pregnancies confirms the usefulness of immunohistochemistry and cytometry but demonstrates the importance of the combination of both techniques to assist histology for the best reliable diagnosis.

## Introduction

Early spontaneous abortion represents 15% of all known pregnancies (Wilcox et al. 1988; Lorenzato et al. 1995). Pathologies related to these pregnancies lead to the problem in diagnosing between complete mole, partial mole and hydropic abortion. (Van Lijnschoten et al. 1993). The clinical impact of these diagnoses is important regarding the occurrence of persistent gestational trophoblastic disease (GTD) after complete mole (CM) (10%–20%) and partial mole (PM) (0.5%–6.6%) (Bagshawe et al. 1990; Rice et al. 1990; Szulman, 1988).

More challenges are experienced nowadays with increased number of prenatal screening techniques providing earlier clinical recognition and termination of abnormal pregnancies (Sebire et al. 2002).

Pathologists are studying most of the products of conception from the first trimester. Differential diagnosis between hydropic changes, PM and CM during this period is more difficult due in part to the persistence of rare collapsed embryonic blood vessels within the chorionic villi in PM as well as in CM (Sebire et al. 2002) and the presence of pseudo cisterns that can be seen in cases of hydropic degeneration.

Furthermore, new entities described in the last years as placental mesenchymal dysplasia associated with high rates of intrauterine growth restriction and fetal demise seems to interfere with molar disease diagnosis (Chan and Sampson, 2003; Gibson et al. 2004); Pham et al. 2006; Parveen et al. 2007; Kinoshita et al. 2007).

Thus the importance for pathologists to improve their diagnostic experience in this new situation and find new ancillary techniques useful for differentiation of early lesions. Van Lijnschoten et al. have determined different histological items arranged in groups called general, stromal, trophoblastics characteristics and intervillous space (Van Lijnschoten et al. 1993). Lorenzato et al. have considered the following histological features: hydropic swelling of the villi, presence of cisterns, villous scalloping, trophoblastic cells in villous stroma, trophoblastic inclusions, micro calcifications and fibrosis (Lorenzato et al. 1995). Sebire et al. have described in 2002 what are the most reliable histological villous abnormalities seen in abnormal conceptions (Sebire et al. 2002). Some details may vary according to the different authors, but the most important histological criteria have been accepted by most of them. However, the criteria sometimes overlap or are subjective showing a considerable inter and intraobserver variability (van Lijnschoten et al. 1993; Fukanaga et al. 2004). In order to discriminate the largest part of the cases, some ancillary methods have been proposed like DNA quantification that can be useful in detecting aneuploidies (Lage et al. 1992; Jeffers et al. 1995).

Recently, a cell cycle inhibitor and tumor suppressor protein p57^kip2^ encoded by a strongly paternally imprinted gene has been explorated as a diagnostic marker in hydatidiform mole, under expressed in CM but not in PM and HA (Lee et al. 1995; Chilosi et al. 1998; Castrillon et al. 2001; Fukunaga, 2002).

The aim of our study was to evaluate on our collective of cases the efficacy of the different ancillary techniques proposed in the literature for difficult situations of differential diagnosis in early pregnancies between HA and PM, PM and CM and HA and CM.

## Material and Method

Paraffin-embedded gestational products from 111 patients, including 28 CM, 23 HA and 60 PM, were collected for this study. All materials received at the Department of Pathology, University of Lausanne, Switzerland obtained by abortion or suction curettage was evaluated. The age of the products of conception varied between 6 and 17 weeks. The age of the mothers ranged from 17 to 43 years. Some cases were related to known cytogenetic abnormal chromosomal content (trisomy 21, 18, or 13), others were collected after early abortion or non-growing pregnancy.

All retrospective material was selected from our files according to the rules of our institution.

## Histological Parameters

Two pathologists reviewed all specimens. The histological criteria considered for this analysis were defined according to the main diagnostic histopathological features and differences described by Sebire et al. 2002. The following histological criteria were considered for diagnosis: villous size, outlines, stroma and hydrops; cistern formation, stromal karyorrhectic debris, villous vessels, trophoblast pseudo inclusions, trophoblast hyperplasia, extra villous trophoblast, implantation site.

## DNA-Ploidy Analysis

One representative paraffin block from all 111 cases was chosen for DNA cytometry analysis (static cytometry). This technique was preferred to flow cytometry, in order to obtain the most adequate material for the analysis using a micro dissection of the paraffin block in separating placental villi from the decidual tissues. Furthermore, the availability of this image processing analysis allows the observer direct visualization of the cell population being studied for DNA measurement, providing a simultaneous correlation between DNA cell content and morphology. 50 μm thick paraffin tissue sections were cut for the analysis and a last 5 μm thick haematoxylin and eosin stained section was prepared for histological control. Nuclear suspensions from the material were prepared using the method of Hedley (Hedley et al. 1983). The suspensions were cytocentrifuged on to glass slides. Two cytospin slides were prepared for each case. For Feulgen staining (Hedley et al. 1983), the slides were first hydrolysed in 5N hydrochloric acid at 25 °C for 45 min. They were then stained with Schiff reagent for 60 min. Sulfite rinse was used. They were rinsed and dehydrated in subsequently changes of alcohol 50%, 70%, ethanol alcohol and xylene for 3 min each before being coverslipped using Eukitt mounting medium. DNA analysis was performed on Feulgen-stained cytospins using an image analysis system Autocyte Quic DNA, version 1.1 (Autocyte Inc., Burlington, NC. A minimum of 150 nuclei was measured per analysis (300 nuclei for most cases). Only well preserved nuclei were selected by the operator. As control we used human lymphocytes (external reference) and tissue polymorphonuclear leucocytes when available (internal reference). The moles were classified according to the DI of the main peak (Barclay et al. 1993). Histograms exhibiting a G0/G1 main peak at 2C were considered to be diploid (index 0.85–1.15). A sample was considered tetraploid when a main peak was found between 1.70 and 2.30 comprising. Similarly, a triploid sample was defined by an index between 1.3 and 1.55. All the measurements were performed by the same qualified cytotechnician.

## Immunohistochemistry

For p57^kip2^ immunohistochemistry reaction, four- μm thick tissue sections were mounted on aminopropylmethoxysilane-coated glass slides, deparaffinized in xylol, taken through to absolute alcohol, blocked for endogenous peroxidase with 0.1% hydrogen peroxide in methanol (45 minutes) and rehydrated through graded alcohols. They were boiled for 15 minutes in 10 mM citrate buffer, pH 6.0 (microwave oven) and rinsed in Tris-buffered saline (TBS; Tris 0.05 M, NaCl 0.9%, pH7.6). To reduce nonspecific binding, they were incubated for 10 minutes in normal horse serum (Pel-Freez Biologicals, Rogers, AK) 1:30 in TBS. After 40-minutes incubation with a prediluted anti-p57^kip2^ mouse monoclonal antibody (Clone 57P06, NeoMarkers, Fremont, CA), the sections were incubated for 30 minutes with biotinylated horse antimouse immunoglobulins (Vector, Burlingame, CA) diluted 1:400 in TBS containing 2% bovine serum albumin, then for 30 minutes with ABC-peroxidase complex solution (Vector) prepared according to the manufacturer’s instructions. Peroxidase activity was revealed with 5-5’-diaminobenzidine as chromogen and the sections were counterstained in Mayer’s acid-free hematoxylin. As negative control, the primary antibody was replaced by a mouse hybridoma supernatant of similar isotype (IgG2b).

## Results

From the 111 cases analyzed, 28 cases corresponded histologically to a complete mole, 60 cases were partial moles and 23 cases taken as control were hydropic abortus. All 111 cases were analysed by image DNA cytometry and immunohistochemistry p57^kip2^.

### Hydropic abortus (Fig.1)

All hydropic abortus cases presented a diploid DNA content and p57^kip2^ revealed positive in the cytotrophoblast and negative in the syncythiotrophoblast. The gestational age ran between 8 to 15 weeks (mean age: 10 weeks). The mean patient age was 34,6 years.

### Complete moles (Fig. 2)

From the 28 complete mole cases, 12 (43%) were diploid and 16 (57%) were tetraploid for DNA analysis but all showed negative cytotrophoblast and syncythiotrophoblast for p57^kip2^ with clear positive internal controls.

The gestational age was comprised between 6 and 12 weeks with a mean gestational age of 10.6 weeks for diploid moles and 8.4 weeks for tetraploid moles. The patients with tetraploid moles were older (mean: 30.4 years; range: 27–36 years) compared to the patients with diploid moles (mean: 27.3 years; range: 19–31 years).

### Partial moles (Fig. 3)

From the 60 PM, 58 cases showed a clear reactivity in the cytotrophoblast for p57^kip2^ staining. Two cases revealed negative for p57^kip2^ staining with positive internal controls, but one case was triploid for DNA nuclear content analysis and the second was tetraploid.

From the 60 cases positive for p57^kip2^ marker, 53 cases revealed triploid (90%), 6 cases were tetraploid (10%) and one case (2%) showed a diploid nuclear DNA content.

The maternal age was comparable between the three groups of PM, however the tetraploid group was younger ( mean age: 29.6 years for triploid PM; 27.8 years for tetraploid PM and 31.5 years for diploid PM). The gestational age was comprised between 7 and 17 weeks ( mean age: 16.7 weeks for triploid PM, 9 weeks for tetraploid PM and 10 weeks for diploid PM).

## Discussion

The need for a pathologist to follow standardized diagnostic criteria to manage material from early spontaneous abortion has resulted in an abundant literature describing different aspects of trophoblastic diseases. If all authors agree on the risk of molar disease to developing persistent gestational trophoblastic tumours as choriocarcinoma or placental site trophoblastic tumour (Bagshawe et al. 1990; Rice et al. 1990; Cui et al. 2004; Nieman et al. 2007) and most of them have emphasized the importance of some ancillary techniques as cytometry and immunohistochemistry to improve diagnosis (Lage, Driscoll et al. 1989; Hitchkock et al. 1991; Lage et al. 1992; Barclay et al. 1993; Coran et al. 1993; Jeffers et al. 1993; Van der Kaa et al. 1993; Carey, 1994; Berezowsky et al. 1995; Jeffers et al. 1995; Lee et al. 1995; Paradinas et al. 1996; Castrillon et al. 2001; Genest et al. 2001; Fukunaga, 2002), heterogeneity of the results from the different studies has raised several questions about how to interprete different data. As well histological criteria for diagnoses of molar diseases had to be changed with earlier evacuation of gestational material (Sebire et al. 2002), as well unusual data in cytometry analyses have disturbed the classical interpretation proposed for diagnosis of partial or complete moles. The straightforward hypothesis that partial mole is triploid and complete mole is diploid is nowadays compromised by different outcomes that reveal an important percentage of tetraploid complete moles but also the evidence of some tetraploid or even diploid partial moles (Surti et al. 1986; Lage, Weinberg et al. 1989; Toth et al. 1992; Bewtra et al. 1997; Lembet et al. 2000, Crisp et al. 2003) .

In our study we intended to determine the most reliable protocol able to achieve the optimal diagnosis in this particular complicated pathology.

After reviewing histopathological criteria according to the description from Sebire et al. 2002, we have collected 28 CM, 60 PM and 23 HA. As HA were chosen to play the role of control for our techniques, all cases were diploid for DNA nuclear content analysis and p57^kip2^ positive. The group of CM revealed in part tetraploid moles (57%) and in part diploid moles (43%) as already mentioned by Bewtra et al. 1997. As these authors precised, we have also observed that tetraploid CM occur in older patients and at lower gestational age. But ten years after their report, although the age of patients with tetraploid CM seems to be comparable, the age of the patients with diploid CM has markedly increased. On the contrary, for both situations the gestational age is lower (8.4 wks and 10 wks vs 12.7 wks and 15.4 wks). However, there was no difference for immunohistochemistry and all these CM were negative for p57^kip2^ staining confirming the diagnosis of CM. More difficult to discuss are the PM which resulted for most of them triploid for DNA nuclear content but 6 cases were tetraploid, a surprising result already described in 1992 by Toth et al. 1992. 5 from these 6 cases were positive for p57^kip2^ confirming their diagnosis of PM. The last case was negative for p57^kip2^ staining suggesting a possible wrong orientation of the histological diagnosis (PM instead CM). Immunohistochemistry was also positive for p57^kip2^ in one case histologically described as PM but revealing a diploid DNA content. Finally, 1 case of PM was normally triploid but presented a negative staining for p57^kip2^. To our knowledge, rare are the cases in which some triploid cases revealed negative for p57^kip2^ with internal positive controls (decidual cells corresponding to the maternal component and always p57^kip2^ positive). The loss of expression of p57^kip2^ in the cytotrophoblast demonstrates the presence of paternal genome with absence of a maternal contribution and is observed generally in CM. Some exceptions have been reported by Fukunaga in 2002. In his paper, he described that p57^kip2^ expression was not found in 4 of 20 HAs and 1 PM although some decidual cells showed the positivity. This could possibly be a false-negative result due to loss of antigenity and diagnosis of partial mole is ensured by the presence of a triploid DNA content. But these exceptions show that immunostaining is certainly important in the management of molar diseases but must be carefully interpreted in conjunction with clinical informations, histopathologic features and ploidy analyses.

In conclusion, no single technique can be used to assist histology for the diagnosis of a molar disease (Crisp et al. 2003). Ploidy results are diploid for complete mole as well as hydropic abortus, sometimes tetraploidy is found in partial and complete moles, p57^kip2^ immunohistochemistry may be positive in partial mole as well as in non-molar miscarriage. But the combination of histology, cytometry and immunohistochemistry remains more reliable protocol to obtain the most correct diagnosis in this type of pathology.

## Figures and Tables

**Figure 1.**
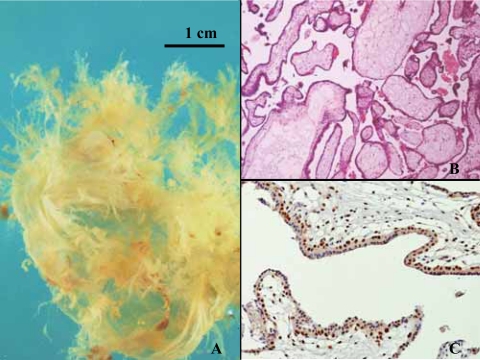
Hydropic abortus. (**A**) Macroscopical view. Product of conception photographed in water. Thin villi are seen. (**B**) Histology, HE staining, X40. Few enlarged villi with scanty stroma. (**C**) p57^kip2^ immunostaining, ×100. Strong positive cytotrophoblastic cells in a linear distribution.

**Figure 2.**
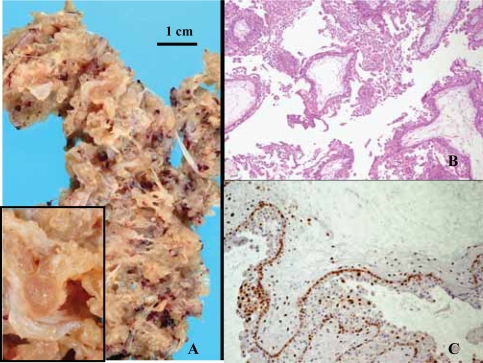
Partial hydatidiform mole. (**A**) Macroscopical view. Well developped villous vesicules (see insert) against a background of smaller vesicles or non-expanded villi. (**B**) Histology, HE staining, X40. Note large villi with syncithiotrophoblastic hyperplasia and confluence of loculated edematous foci with a well-formed cistern in the middle. (**C**) p57^kip2^ immunostaining, ×100. Strong positivity of cytotrophoblastic cells.

**Figure 3.**
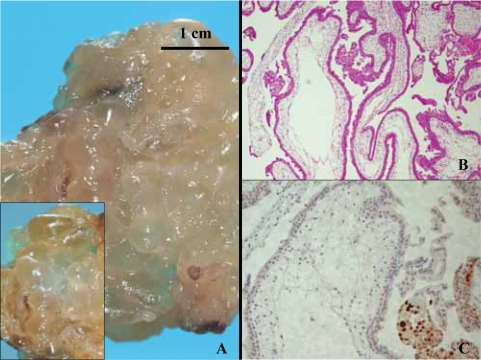
Complete hydatidiform mole. (**A**) Macroscopical view. « Bunch of grapes » group of hydatidiform villi with large vesicles, most of them representing end-stage cistern. (**B**) Histology, HE staining, X40. Note generalized edema leading to cistern formation. Trophoblastic hyperplasia is marked. (**C**) p57^kip2^ immunostaining, ×100. syncithio and cytotrophoblastic cells are negative in the villi. Internal immunostaining (decidual cells)* control is strongly positive.
